# Plasmapheresis in a patient with antiphospholipid syndrome before living-donor kidney transplantation: a case report

**DOI:** 10.1186/1471-2369-15-167

**Published:** 2014-10-15

**Authors:** Tadashi Sofue, Yushi Hayashida, Taiga Hara, Kazuyo Kawakami, Nobufumi Ueda, Yoshio Kushida, Masashi Inui, Hiroaki Dobashi, Yoshiyuki Kakehi, Masakazu Kohno

**Affiliations:** Division of Nephrology and Dialysis, Department of CardioRenal and Cerebrovascular Medicine, Faculty of Medicine, Kagawa University, 1750-1 Ikenobe, Miki-cho, Kita-gun, Kagawa, 761-0793 Japan; Department of Urology, Kagawa University, Kagawa, Japan; Department of Pathology, Kagawa University, Kagawa, Japan; Department of Urology, Tokyo Women’s Medical University, Yachiyo Medical Center, 477-96 Owada-Shinden, Yachiyo, Chiba 276-8524 Japan; Division of Endocrinology and Metabolism, Hematology, Rheumatology and Respiratory Medicine, Department of Internal Medicine, Kagawa University, Kagawa, Japan

**Keywords:** Antiphospholipid syndrome, Living-donor kidney transplantation, Plasmapheresis, Anti-β2-glycoprotein I IgG

## Abstract

**Background:**

Early graft thrombosis and bleeding complications remain important causes of early graft loss following kidney transplantation in patients with antiphospholipid syndrome. Anti-β2-glycoprotein I IgG is a disease-specific antibody in patients with antiphospholipid syndrome. Although plasmapheresis is partially effective for antibody removal, the optimal treatment allowing successful transplantation in patients with antiphospholipid syndrome has not been established. This is the first report of a patient with antiphospholipid syndrome who successfully underwent living-donor kidney transplantation following prophylactic plasmapheresis for removal of anti-β2-glycoprotein I IgG.

**Case presentation:**

A 37-year-old Japanese female was scheduled to undergo a living-donor kidney transplant from her mother. At age 25 years, she experienced renal vein thrombosis, was diagnosed with antiphospholipid syndrome secondary to systemic lupus erythematosus, and was subsequently treated with prednisolone and warfarin. At age 37 years, she was diagnosed with end stage kidney disease, requiring maintenance hemodialysis because of recurrent renal vein thrombosis despite taking anticoagulation therapy. The pretreatment protocol consisted of prophylactic plasmapheresis plus full anticoagulation therapy to counteract the risks of early graft thrombosis. Anticardiolipin and anti-β2-glycoprotein I IgGs were successfully removed by both double filtration plasmapheresis and plasma exchange. The allograft kidney began to function soon after transplantation. No obvious thrombotic complications were observed after transplantation, although anti-β2-glycoprotein I IgG increased to the level observed before plasmapheresis. One year after transplantation, the patient’s kidney function remains stable while receiving anticoagulation therapy as well as a maintenance immunosuppressive regimen.

**Conclusion:**

Prophylactic plasmapheresis plus full anticoagulation therapy may be an effective strategy in patients with antiphospholipid syndrome undergoing living-donor kidney transplantation.

## Background

Early graft thrombosis and bleeding complications remain important causes of early graft loss following kidney transplantation in patients with antiphospholipid syndrome (APS) [1–4]. APS is a multisystem autoimmune disorder characterized clinically by recurrent arterial and/or venous thrombosis and/or pregnancy morbidity, and serologically by the presence of antiphospholipid antibodies (aPL), including lupus anticoagulant (LA), anticardiolipin (aCL) and anti-β2-glycoprotein I (anti-β2GPI) antibodies. APS can be classified as primary or secondary to systemic lupus erythematosus (SLE). aCL antibody has been regarded as a major antiphospholipid antibody and a marker for APS. In contrast, anti-β2GPI antibody is a relatively new disease-specific antibody for APS and considered a cause of thrombotic complications [5, 6].

The “two hit” model of thrombosis in APS patients states that an initiating “first hit” injury disrupts the endothelium, and that aPL potentiates thrombus formation as a “second hit” [7]. The “first hit” injury to the endothelium can include trauma, surgery, infection and drugs [8, 9], making surgical procedures an important risk factor for thrombosis in patients with APS.

Anticoagulation therapy with warfarin is recommended in patients with APS to prevent recurrent arterial and/or venous thrombosis [10, 11]. Anticoagulation therapy before and at the time of kidney transplantation has been reported to reduce the risk of early posttransplant thrombosis in the allograft [2]. However, anticoagulation therapy increases the risk of bleeding complications, which may lead to early allograft loss [3]. Moreover, patients with APS are at high risk of allograft thrombosis even when taking anticoagulation therapy [3].

Prophylactic temporary plasmapheresis for antibody removal has been reported effective in patients with APS and acute thrombotic complications [12, 13]. Plasmapheresis has been found to reduce serum titers of aCL and anti-β2GPI IgGs [12, 14]. Although anticoagulation therapy with heparin is recommended for pregnant women with APS [15], prophylactic plasmapheresis has been reported partially effective in these women [16, 17]. To our knowledge, prophylactic plasmapheresis has been reported useful in only one patient with primary APS undergoing kidney transplantation [4]. Therefore, the optimal treatment strategy allowing successful kidney transplantation in patients with APS has not yet been established. This report describes a patient with secondary APS, who underwent prophylaxis with plasmapheresis, in addition to full anticoagulation therapy, prior to successful living-donor kidney transplantation.

## Case presentation

A 37-year-old Japanese woman, scheduled to undergo a living-donor, ABO-compatible kidney transplant from her mother, was referred to our kidney transplant center in July 2012. At age 25 years, she had experienced acute kidney injury (AKI) due to bilateral renal vein thrombosis. At that time, she was diagnosed with APS secondary to SLE because of the repeated detection of high titers of antibody to double-stranded DNA (300 U/ml) and aCL IgG (39.6 U/ml) and low serum complement 3 (C3; 34 mg/dl) and complement 4 (C4; 2 mg/dl) concentrations. Following the diagnosis of AKI, she underwent hemodialysis (HD) for 2 weeks. Plasma exchange (PE), methylprednisolone pulse therapy and anticoagulation therapy ameliorated her AKI, and she no longer needed HD. She subsequently received immunosuppressive therapy with 5 mg prednisolone and anti-coagulant therapy with warfarin, to maintain an international normalized ratio (INR) between 2.0 and 3.0. During this time, her serum creatinine concentration remained stable (1.2 to 1.4 mg/dl; 103 to 120 μmol/L). However, at age 36 years, she experienced recurrent bilateral renal vein thrombosis and was again diagnosed with AKI. At the time of recurrent thrombosis, her INR was 1.5, which was lower than the target therapeutic range, despite receiving a sufficient amount of warfarin (3.0 mg/day). At the time of recurrence, her LA was positive (ratio 1.3; normal range <1.3), as determined by the Gradipore-LA Dilute Russell’s viper venom time (dRVVT) test (Medical & Biological Laboratories Co., Ltd., Nagoya, Japan). The combination of immunosuppressive and anticoagulant therapy could not restore her kidney function. At age 37 years, she was diagnosed with end stage kidney disease, requiring maintenance HD. Because of thrombosis, three consecutive operations for arteriovenous fistula failed. Finally, an arteriovenous graft was created as vascular access for HD. All tests performed to investigate the causes of thrombophilia were within normal ranges, except for aPL assays. SLE activity was considered low because her serum C3 (91 mg/dl) and C4 (23 mg/dl) concentrations were normal, and there was no evidence of antibody to double-stranded DNA (<5.0 IU/ml). Her pre-treatment aCL and anti-β2GPI IgG concentrations were 42.6 U/ml (normal range <10 U/ml) and 13.9 U/ml (normal range <3.5 U/ml), respectively. Plasma aCL IgG (MESACUP cardiolipin test, Medical & Biological Laboratories Co., Ltd) and anti-β2GPI IgG (anti-CL-β2GPI EIA kit, Yamasa Co., Choshi, Japan) were measured by enzyme-linked immunosorbent assays. The dRVVT test before kidney transplantation was positive (ratio 1.3). Her 63-year-old mother had sufficient eGFR (109 ml/min/1.73 m^2^) and two human leukocyte antigen (HLA) mismatches with the recipient. Tests for histocompatibility, including complement-dependent cytotoxicity (CDC) crossmatch, flow crossmatch and flow cytometry panel-reactive antibody (PRA) tests, showed that the donor and recipient were histocompatible.

Kidney transplantation was planned for February 2013. The pretreatment protocol consisted of prophylactic plasmapheresis plus full anticoagulation therapy to counteract the risks of early graft thrombosis and bleeding. Double filtration plasmapheresis (DFPP) was performed using a KM-8900EX (Kuraray Medical, Tokyo, Japan) machine with a Plasmacure PE-05 plasma separator (Kuraray Medical) and Evaflux 2A20 plasma fractionator (Kawasumi Laboratories, Tokyo, Japan). During DFPP, 2500 ml of blood was treated, with replacement by 450 ml of 11.25% albumin solution. PE was performed using a KM-8900EX machine with a Plasmacure PE-05 plasma separator, with replacement by 2700 ml of fresh frozen plasma (FFP).

**Figure 1:**
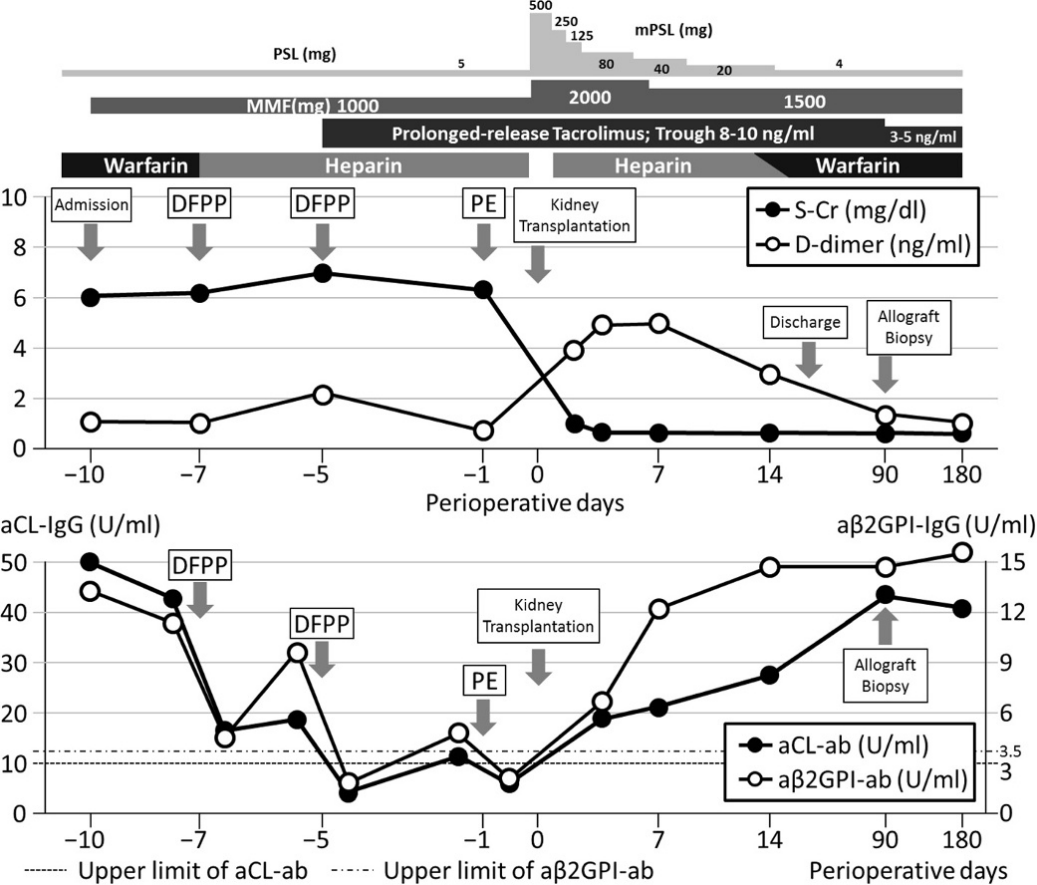
**Clinical course and treatment of the patient.** Abbreviations: PSL, prednisolone; mPSL, methylprednisolone; MMF, mycophenolate mofetil; S-Cr, serum creatinine; DFPP, double filtration plasmapheresis; PE, plasma exchange; aCL, anticardiolipin; IgG, immunoglobulin G; aβ2GPI, anti-β2 glycoprotein I.

The clinical course and treatment of the patient are shown in Figure 1. The induction immunosuppressive regimen consisted of combination therapy with prolonged-release tacrolimus, mycophenolate mofetil (MMF), methylprednisolone and basiliximab. MMF (1000 mg/day), was started 10 days before kidney transplantation. Anticoagulation therapy with 2.5 mg/day warfarin was replaced by daily continuous injections of heparin (10,000 units/day), starting 7 days before transplantation, with the dose of heparin adjusted to maintain an activated partial thromboplastin time (APTT) of 40 to 60, with heparin suspended 3 hours before surgery. DFPP was performed on days -6 and -4 and PE on day -1. aCL and anti-β2GPI IgGs were successfully removed by both DFPP and PE, with both being within normal range on day -1 (3.8 IU/ml and 1.9 U/ml, respectively). No apparent adverse effects were observed during plasmapheresis.

The right kidney of the donor was transplanted into the right iliac fossa of the recipient. Intraoperative bleeding was 582 ml and the amount of transfused red cell concentrates was 560 ml. Post-operative hemoglobin was 9.4 g/dl. Allograft kidney function rapidly appeared after kidney transplantation. Anti-coagulation therapy with heparin (10,000 units/day) was restarted on the morning of day +1 and replaced by warfarin on day +14. After transplantation, aCL and anti-β2GPI IgGs increased to their levels before plasmapheresis. However, no obvious thrombotic complications, including increased serum d-dimer levels, were observed after transplantation. The patient was discharged 21 days after transplantation, receiving maintenance anti-coagulation therapy with warfarin (3 mg/day) and a maintenance immunosuppressive regimen, consisting of prolonged-release tacrolimus (6 mg/day), MMF (1500 mg/day) and methylprednisolone (4 mg/day).

**Figure 2:**
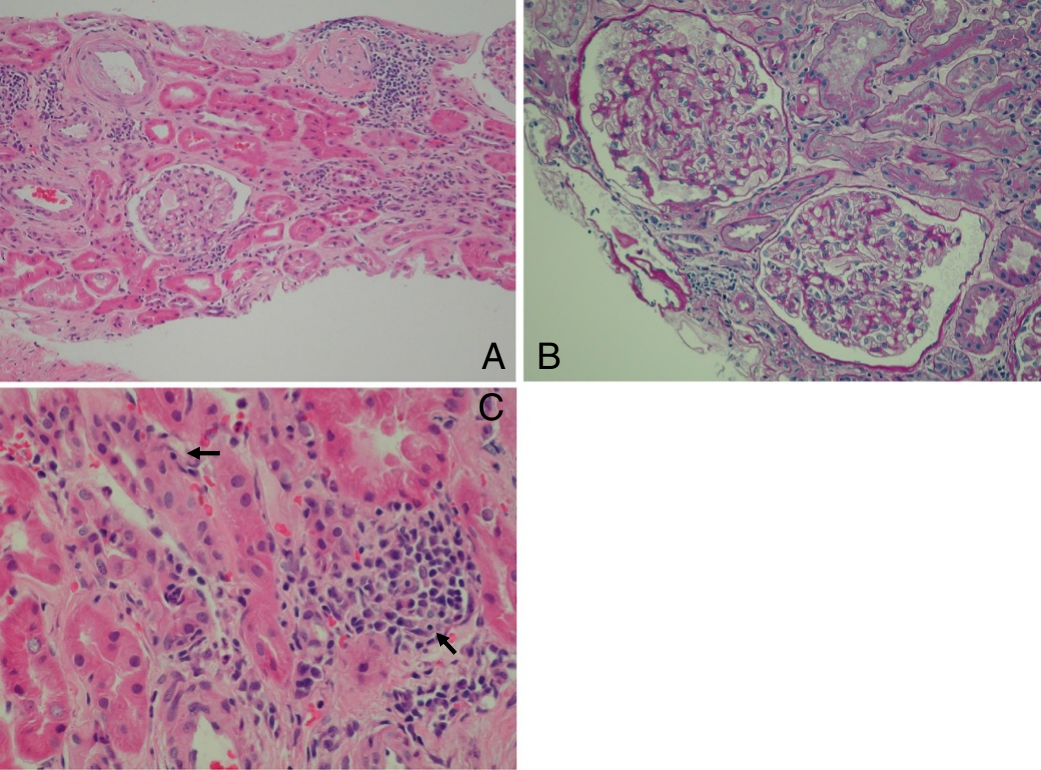
**Renal histopathological findings showing the absence of (A) arteriole thrombosis (hematoxylin and eosin staining, low power field) and (B) lupus nephritis (periodic acid-Schiff staining, high power field), but (C) slight lymphocyte infiltration into tubules (Banff score, t1) (hematoxylin and eosin staining, high power field).**

Three months after kidney transplantation, her serum creatinine level was stable (0.69 mg/dl; 59.5 μmol/L) without apparent proteinuria, and her titer of anti-β2GPI IgG was high (17.8 U/ml). Pathological findings of a protocol allograft biopsy taken 3 months after kidney transplantation are shown in Figure 2. There was no evidence of arteriole thrombosis or lupus nephritis. Her Banff score was t1 i1 g0 v0 ci0 ct1 mm0 cv0 ah0 ptc0; and immunofluorescence findings were negative. Borderline changes were diagnosed.

At the present time, one year after transplantation, her serum creatinine concentration remains stable (0.67 mg/dl; 57.8 μmol/L), although she has a high anti-β2GPI IgG titer (31.9 U/ml) and is positive on the dRVVT test (ratio 1.3). She has not experienced any bleeding or thrombotic complications while remaining on anticoagulant therapy with warfarin to maintain an INR between 2.0 and 3.0.

## Discussion

We have shown here that prophylaxis with plasmapheresis and full anticoagulation therapy was an effective strategy in a patient with secondary APS who underwent living-donor kidney transplantation. These treatments are important because once the thrombotic process is initiated, there is no effective therapy available to lyse the clot and to recover the allograft [3].

Prophylactic plasmapheresis could reduce both aCL and anti-β2GPI IgGs to their normal ranges. Although we found that DFFP removed 70% of both aCL and anti-β2GPI IgGs, a previous report found that DFPP with Evaflux 2A20 removed around 20% of total IgG [18]. We also found that PE removed 56% of both aCL and anti-β2GPI IgGs, whereas product information for Plasmacure PE-05 stated that PE removed 96% of total IgG [19]. Treatment with PE has been reported to reduce anti-HLA antibodies while not affecting tetanus IgG in kidney transplant recipients [20]. As replacement with FFP should be effective in maintaining permanent immunity, we considered that our method of plasmapheresis could remove disease-specific IgG while leaving permanent immunity intact. Unfortunately, however, we could not determine whether plasmapheresis affected the results of dRVVT tests. A case report showed that treatment with plasmapheresis decreased dRVVT test results [21]. Thus, IgG removal by plasmapheresis would remove LA as well as other aPLs.

We used prophylactic temporary plasmapheresis in this patient only to prevent perioperative thrombotic complications. Endothelial injuries can result from the surgical procedures used during kidney transplantation, including perfusion with organ preservation solution and ischemia/reperfusion injury, although these injuries are considered temporary. Therefore, temporary prophylactic aPL removal may have reduced the risk of thrombosis during the early post-operative period. Moreover, perioperative bleeding complications may be prevented by discontinuing anticoagulant therapy. That is, removal by plasmapheresis of a sufficient amount of anti-β2GPI IgG could allow anticoagulant therapy to be safely discontinued for 24 hours perioperatively. Although the maintenance immunosuppressive regimen could not reduce anti-β2GPI IgG after kidney transplantation, continuous plasmapheresis to remove increased anti-β2GPI IgG was not recommended. An international consensus statement has suggested that plasmapheresis be used only at the time of thrombotic complications [10]. Sufficient anticoagulation therapy during the maintenance phase after kidney transplantation is considered effective in preventing thrombotic complications in patients with APS.

A recent non-randomized pilot study showed that rituximab, a chimeric monoclonal antibody against CD20, may play a role in the treatment of patients with refractory catastrophic APS [22]. In Japan, rituximab is frequently used as an induction immunosuppressant for ABO-incompatible kidney transplantation [23]. However, treatment with rituximab was unable to change aPL profiles [24]. Moreover, two randomized clinical trials with rituximab (the EXPLORER and LUNAR studies), which did not show rituximab efficacy in patients with SLE, did not include patients with APS [25, 26]. Because we considered the efficacy and safety of rituximab in patients with APS secondary to SLE not established, we did not treat our patient with rituximab prophylaxis.

The long-term prognosis of patients with APS after kidney transplantation has been reported to be poor [27]. Although these patients did not develop major thrombotic complications, the presence of aCL IgG was associated with poor transplantation outcomes [27]. Recently, anti-β2GPI IgG was reported to initiate arteriosclerotic changes [6]. Long-term monitoring of cardiovascular disease and kidney function is necessary for these patients.

## Conclusions

We describe here the successful living-donor kidney transplantation in a patient with secondary APS following a combination of prophylactic plasmapheresis and anticoagulation therapy. Although it is impossible to conclude that plasmapheresis is useful as prophylaxis for thrombosis in patients with APS undergoing kidney transplantation based on the findings in a single patient, our results suggest that further studies are warranted.

## Consent

Written informed consent was obtained from the patient for publication of this Case report and any accompanying images. A copy of the written consent is available for review by the Editor of this journal.

## Abbreviations

aCL: Anticardiolipin
AKI: Acute kidney injury
aPL: Antiphospholipid
APS: Antiphospholipid syndrome
APTT: Activated partial thromboplastin time
β2GPI: β2-glycoprotein I
C3: Complement 3
C4: Complement 4
CDC: Complement-dependent cytotoxicity
DFPP: Double filtration plasmapheresis
dRVVT: Dilute Russell’s viper venom time
FFP: Fresh frozen plasma
HD: Hemodialysis
HLA: Human leukocyte antigen
INR: International normalized ratio
LA: Lupus anticoagulant
MMF: Mycophenolate mofetil
PE: Plasma exchange
PRA: Panel-reactive antibody
SLE: Systemic lupus erythematosus.
